# Photocarrier Recombination Dynamics in Highly Scattering Cu_2_O Nanocatalyst Clusters

**DOI:** 10.1021/acs.jpcc.3c06941

**Published:** 2024-01-24

**Authors:** Sunil Gyawali, Ravi Teja A. Tirumala, Harrison Loh, Marimuthu Andiappan, Alan D. Bristow

**Affiliations:** † Department of Physics and Astronomy, West Virginia University, Morgantown, West Virginia 26506, United States; ‡ School of Chemical Engineering, Oklahoma State University, Stillwater, Oklahoma 74078, United States; § Department of Mechanical and Aerospace Engineering, West Virginia University, Morgantown, West Virginia 26506, United States

## Abstract

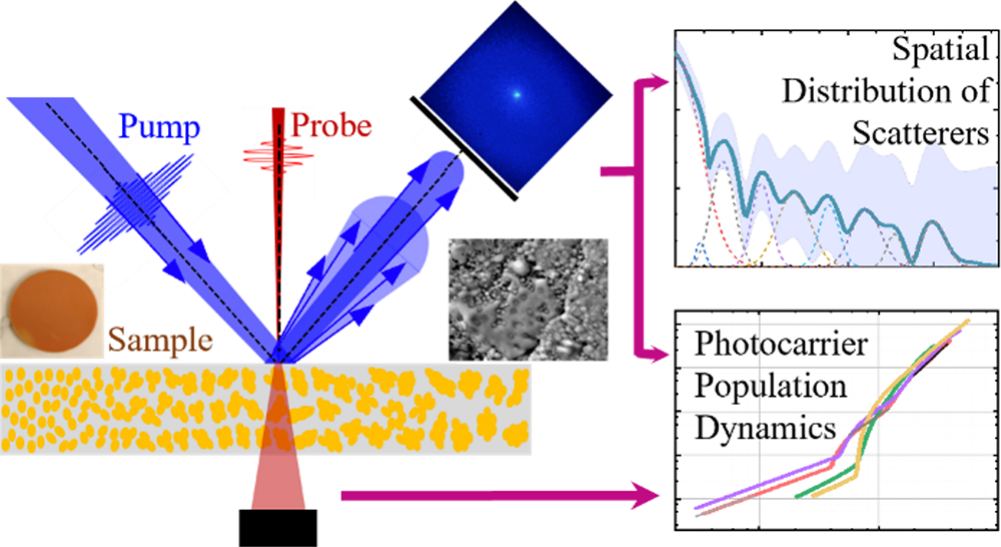

Inversion analysis of transient absorption data to capture the photoexcited charge carrier population rate dynamics is a powerful technique for extracting realistic lifetimes and identifying recombination pathways. However, for highly scattering samples such as Cu_2_O nanoparticles (NPs) with associated dielectric Mie scattering, the scattering leads to an inaccurate measure of the excited photocarrier. This work studies methods to correct for the scattering to generalize the use of inversion analysis and provide secondary information about the nature of the scattering NPs. Scattering profiles of semitransparent disks containing Cu_2_O NPs with different shapes and sizes are measured to demonstrate that the inclusion of scattering in analysis reduces the photoexcited carrier density by 1 order of magnitude. It is found that the photocarrier density response is affected by shape rather than size. A Fourier transform of the scattering profiles produces a distribution of length scales within the sample characteristic of the mean separation of scatterers. This analysis reveals that NPs are forming clusters. Links are made between the scattering and carrier dynamics.

## 1 Introduction

Metal-oxide semiconductors are used in applications from thin-film transistors^1^ and optoelectronic devices,^2^ to gas sensors,^3^ and photocatalysts.^4^ The rational design of these materials and their devices requires an understanding of their photon absorption, charge separation, and charge migration processes. Photocarrier dynamics can be measured by transient-absorption spectroscopy,^5−9^ where an optical pump excites photocarriers and that excited-state population is probed with a second pulse arriving after a delay time, adjusted to map out the transient. Transient absorption can determine generic excited-state lifetimes, hot-carrier relaxation mechanisms, and charge-transfer rates. Using the absorption cross-section of the pump radiation, the transients provide an instantaneous carrier concentration as a function of delay time. Differentiating the instantaneous carrier concentration transients recaptures the full rate equation.^10−12^ This inversion analysis method has advantages over global analysis, where the transients only fit with a series of exponential decays because it does not average the information or complete the various simultaneous decay components.

Originally inversion analysis was used to identify Shockley-Read-Hall (SRH) dynamics and bimolecular radiative recombination (RR) in doped silicon.^9^ It has subsequently been used to extract donor-to-acceptor charge-transfer rates in CdS-Au-TiO_2_ sandwich nanorod arrays^10^ and quantum-dot-sensitized-TiO_2_,^11^ measure recombination dynamics from defects states in nitrogen-doped La_2_Ti_2_O_7_,^12^ and determine charge trapping rates in CdSe/Cd_1–*x*_Zn_*x*_S quantum dots.^13^ Inversion analysis has also captured dynamics associated with defects in amorphous g-C_3_N_4_ that hinder photocatalytic H_2_ evolution,^14^ isolated hot-carrier dynamics in type-II band-aligned InAs-based superlattices,^15,16^ identified a reduction of Auger scattering (AS) in InGaN/GaN LEDs through efficient radiative transfer,^17^ and shown that photocarrier dynamics are modified by Mie-resonance scattering in cubic Cu_2_O nanoparticles (NPs).^18^ Additionally, while not fully implemented, aspects of inversion analysis have been applied to population dynamics extracted from two-dimensional electronic spectroscopy,^19^ exchange between separated populations in bulk metal-halide perovskites,^20^ hot-Carrier relaxation in CdSe/CdS Core/Shell nanoplatelets,^21^ carrier-assisted super oxidation in lead-halide perovskites,^22^ transfer kinetics in mixed dye systems,^23^ and the influence of grain boundaries and dopants on the photoluminescence of bulk TiO_2_.^24^

Inversion analysis works well for materials where the absorption coefficient (or cross-section) is well known and the sample is homogeneous and/or highly uniform, such as semiconductor heterostructures grown by molecular beam epitaxy with specular surfaces,^16^ and even nanocrystalline particles,^13^ platelets,^20^ particulate heterostructures,^25^ and hybrid molecular samples,^14^ as long as the absorption, scattering, and extinction cross sections are sufficiently low. In these cases, the incident, reflected, and transmitted pump pulses can be easily measured to determine the number of photons absorbed and the resulting photocarrier density. However, for less ideal conditions, estimates of the excited charge carrier density require a more complete accounting of the photons that interact with the sample. This is especially true in highly scattering samples that are composed of optoelectronically, photonically, or photochemically functional NPs.

In this study, the procedure for analyzing transient absorption to accurately determine the rate equations is performed for highly scattering samples that comprise ≈0.5% Cu_2_O NPs by volume in a dielectric host KBr matrix that is compressed into a semitransparent disk. This material system is chosen because NPs with characteristic dimensions ≳90 nm exhibit strong Mie resonances, perturbed free induction, excited-state lifetimes in excess >1 ns, and enhancement of photocatalytic activity.^18^ The resulting sample is semitransparent for the probe pulse and highly scattered for the pump pulse, leading to potential miscalibration of the carrier concentration and the rate equation. To accommodate the sample properties, measurements of the incident, reflected, transmitted, and scattered pump photons are taken into consideration. The resulting rate curves show that this process can lower the carrier density range by at least 1 order of magnitude, making the carrier concentration consistent with expected transitions from one type of dynamics to another from bulk measurements. In addition to an accurate evaluation of the carrier densities, scattering measurements also provide information about NP clustering. Here, the scattering profile is mathematically transformed to estimate the cluster size and mean separation between clusters. Thus, clustering can be compared to the observed carrier dynamics. The aggregation of NPs is a common phenomenon in many materials and can alter the physical processes, such as electrostatic enhancement and steric repulsion between the NPs, that affect photocatalysis.^26^ Understanding the effects of clustering on light-driven processes can help explain positive impact observed to date.^27−32^

## 2 Experimental Methods

**Figure 1:**
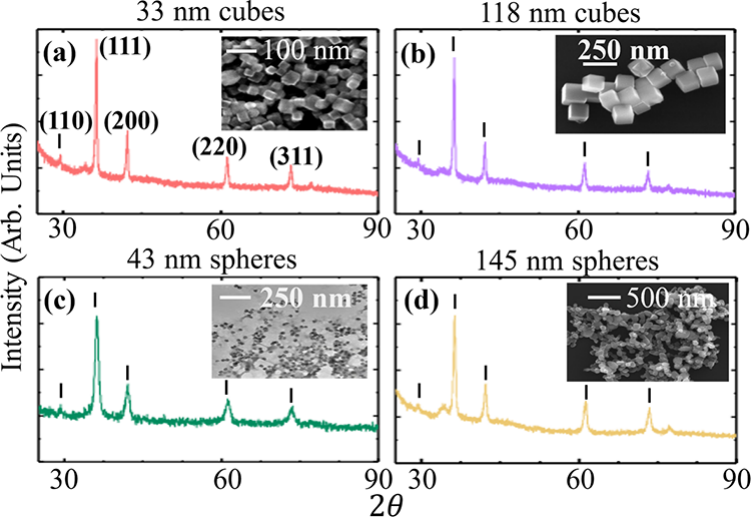
Cu_2_O nanoparticle structural characterization: X-ray diffraction and electron micrographs (inset) of Cu_2_O nanocubes with average edge length (a) 33 nm and (b) 118 nm and nanospheres with average diameters (c) 43 nm and (d) 145 nm. These and all following spectra or transient data are color-coded by sample.

Cu_2_O NPs for this study are produced by the microemulsion technique and chemical reduction method. Microemulsion produce small nanospheres (with average diameters ranging from 35 to 45 nm),^33^ whereas large nanospheres (average diameter of 145 nm) and nanocubes with edge lengths of 90 to 450 nm^34^ are produced by chemical reduction. Detailed procedures for the nanoparticle synthesis are provided in the Supporting Information (SI). Figure 1 shows X-ray diffraction of the synthesized (a) (33 ± 6)-nm and (b) (118 ± 21)-nm nanocubes and (c) (43 ± 5)-nm and (d) (145 ± 41)-nm nanospheres. In each case, the diffraction pattern confirms a high-quality Cu_2_O crystal structure. The inset of each plot shows an electron micrograph for that sample, from which the average shape and size of the NPs are estimated. Images show rounded edges and facets for spherical NPs and distinct corners for the cubed NPs.

**Figure 2:**
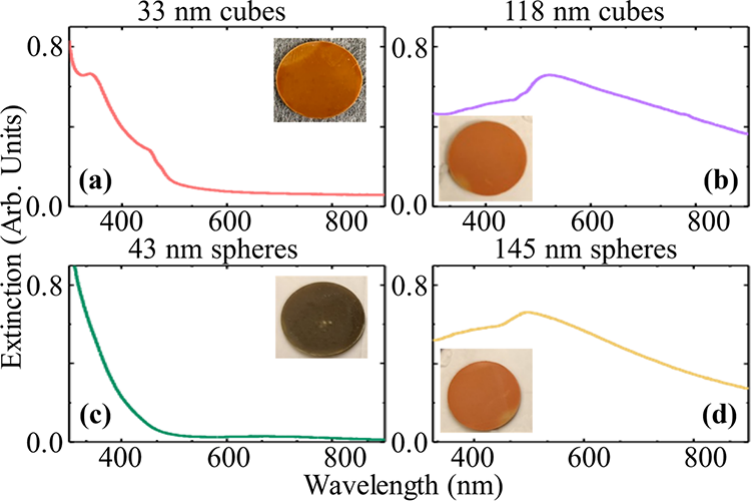
Cu_2_O nanoparticles optical properties – UV–vis extinction spectra of Cu_2_O nanocubes with average edge length (a) 33 nm, (b) 118 nm and Cu_2_O nanosphere with average diameter (c) 43 nm, (d) 145 nm. Insets show photographs of the 1 cm diameter sample disks for the various nanoparticles pressed in a KBr matrix.

Figure 2 shows the extinction spectra for (a) 33 nm and (b) 118 nm nanocubes and (c) 43 nm and (d) 145 nm nanospheres. The experimentally measured spectra in Figure 2 are consistent with the simulated extinction spectra from finite-difference time-domain (FDTD) simulations. These simulated spectra are provided in Figure S1 in the SI. As expected, the 43 nm spheres exhibit extinction spectra that are somewhat similar to their bulk counterparts but with an increased extinction for wavelengths shorter than ≈500 nm due to interband absorption. In contrast, large spheres and cubes show structure in the extinction arising from Mie resonances.^18,34^ These resonances are characterized as small, but significant, peaks at short wavelengths in small cubes and by significant extinction at a much longer wavelength in the larger NPs.

For transient absorption measurements, NPs are mixed with KBr with a concentration of 1% by mass (0.5% by volume) and then pressed under vacuum into semitransparent disks of thickness <0.5 mm. The insets of Figure 2 show photographs of the disks created for each of the four NP samples.

**Figure 3:**
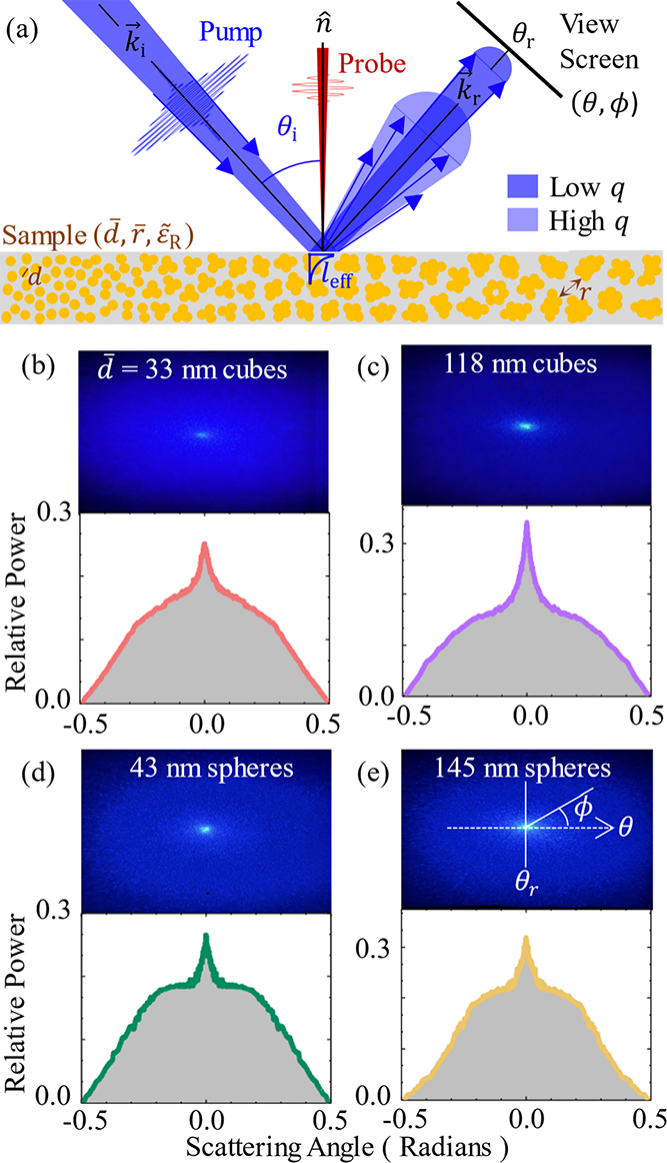
Experimental distribution and scattering profiles: (a) Schematic diagram of the experimental geometry for the transient absorption measurements. Also shown is the sample consisting of different clusters, drawn with an artificial gradient in particle count per cluster from left to right to illustrate the relationship between characteristic parameters, such as the mean separation between clusters (*r̅*), the average nanoparticle size (*d̅*), and resulting mean relative dielectric constant (ε̅_R_). The pump light will have a short effective penetration depth (*l*_eff_), while much of the light is scattered in the reflection direction and measured on a view screen, arranged to have polar coordinates (θ, ϕ). Photographs and the resulting scattering profile are shown for (b) 33 nm, (c) 43 nm, (d) 118 nm, and (e) 145 nm Cu_2_O nanoparticles.

Transient absorption measurements are performed using ≈100 fs pulses with a central wavelength of 800 nm generated by a 1-kHz laser amplifier. The emitted pulses are split into two replicas: one of the pulses is frequency doubled in a β-barium borate crystal to 400 nm to photoexcite all samples with sufficient excess photon energy (*E*_p_ ≈ ℏω_p_ ≈ 3.1 eV); compare to extinction spectra in Figure 2. The second pulse replica is used as a probe and includes a path with a variable relative delay time of up to ≈2 ns. At the sample position, the 1/e^2^ diameter of the pump and probe spots are ≈0.45 and ≈0.1 mm, and the average powers for this experiment are set to ≈1.12 and ≈0.5 mW, respectively. Figure 3a shows a schematic diagram of the transient-absorption geometry at the sample position with the probe at normal incidence–transmitting through the disk and the pump at a non-normal incident angle, such that scattering can be projected onto a view screen, *s* ≈ 14 cm away from the sample, and recorded with a CMOS camera. The pump scattering lobe and the viewing screen are coplanar to each other. Additionally, the average power of the reflected pump scattering is measured by a power meter at ≈3 cm from the sample, so as not to block the incident beams while capturing all the scattered pumps. Power and scatter measurements are performed within an enclosure to screen unwanted background light from the rest of the optical table. Meanwhile, the probe light is transmitted through and loosely focused onto a silicon photodiode, whose signal is fed into a lock-in amplifier that is referenced to a mechanical chopper in the pump path and synchronized to the second subharmonic of the laser repetition rate (250 Hz). While there is scattering of the transmitted probe, it can be ignored because the differential signal measures differences in the total probe intensity reaching the photodetector. Hence, regardless of probe scatter transient absorption results can be directly compared between samples.

## 3 Results and Discussion

### 3.1 Scattering from NP Clusters

Figure 3 shows photographs of the reflected pump scatter on the view screen along with the associated scattering distributions for the NP-KBr disks that consist of (b) 33 nm and (c) 118 nm nanocubes and (d) 43 nm and (e) 145 nm nanospheres. The distributions are determined by taking the average of several line slices captured symmetrically through the center of each photograph using the polar coordinate system (θ, ϕ) illustrated in the photograph of Figure 3e. The polar angle is determined from θ = tan^–1^(*x*/*s*), where *x* is the displacement from the center and *s* is the distance from the sample to the view screen. For each of the four samples, the reflected scatter distribution is azimuthally symmetric about the center, with a brighter central spot at the reflection angle (θ_r_) surrounded by a decaying distribution as the scattering angle (θ) differs from θ_r_. Scattering at θ_r_ is expected to primarily arise from specular reflection over the entire pump beam, which indicates that the surface remains somewhat optically flat. Nevertheless, there is strong scattering away from θ_*r*_ due to the randomness of the Cu_2_O NPs in the KBr matrix.

It should be noted that no forward scattering is observed through the disk, indicating that the sample is optically thick. Since the exact dispersion of the NPs in the disk is random, effective-medium theory applies, and only an effective penetration depth (*l*_eff_) can be assumed for the pump excitation. In this case, *l*_eff_ ≪ *l* is the thickness of the disk. Consequently, it can be assumed that the imaginary part of an effective dielectric function (ε̅_R_) is sufficiently large for the pump scatter to reradiate almost exclusively in the reflection direction. This result contrasts with conventional Mie scattering that produces forward scattering, either from single particles in rarified or optically thin media.^35,36^ Nevertheless, the size of the scatterers means that the pattern remains a result of Mie scattering, which is typically modeled by a series of Bessel functions that can be approximated to Gaussians if there is a strong disorder.^37−39^

The degree of disorder can be evaluated by considering the dispersion of the scattering NPs within the KBr disks. If the NPs are randomly dispersed, individually in the KBr disks, the scattering pattern can be related to their size (characterized by *d̅* for diameter or edge length), and a single mean separation (*r̅*). Given the total mass of Cu_2_O NPs in the KBr matrix is measured to be 1% in all four samples, conversion to the total volume of NPs in each disk is the same because the density of the Cu_2_O is also the same. The volume fraction is Ø = 0.005 = *M V*_NP_/*V*_Disk_, where *M* is the total number of particles and *V*_NP_ and *V*_Disk_ are the volumes of a single NP and the disk. The average NP density, *m* = *M*/*V*_Disk_ = Ø /*V*_NP_, and hence the mean separation are  (more details are given in the SI). For a disk comprising randomly dispersed individual NPs, this would mean that *r̅* ∝ *d̅* and the scattering lobe should be close to a single Gaussian centered around θ_r_. This expectation does not match the observed scattering. For example, the scatter pattern of 33 nm nanocubes has a knee at θ ≈ 0.3 rad, the 118 nm nanocubes exhibit a dome shape, the 43 nm nanospheres have a flat top Gaussian, and the 145 nm nanospheres exhibit a Gaussian tail with a sharp knee at θ ≈ 0.2 rad. Such strong differences in the scatter patterns indicate that *d̅* and/or *r̅* are also distributions, contributing many lobes with different angular widths. Since the NPs have a mild polydispersity quantified by the electron micrographs, this means that *r̅* must have a strong distribution in the disk. This can be satisfied by the clustering of particles, which leads to larger characteristic lengths, *d̅*, and hence *r̅* values. In addition, *r̅* would also scale with the number of particles in a cluster. This is illustrated in Figure 3a, where from left to right in the diagram of the sample, the particle count in the clusters increases along with the mean separation between clusters. For clustered NPs, an estimated volume of the cluster (*V*_C_) can replace *V*_NP_, such that . Table 1 shows a list of estimated *r̅* for small clusters comprising configurations up to six nanospheres of 43 and 145 nm nanospheres, assuming that the nanospheres are in contact.^40^ Beyond six nanospheres per cluster, there is a larger number of touching configurations, and even more partially touching configurations, that for the same particle count yield an ever larger distribution of *r̅* for clusters with the sample particle count; see Figure S3 in the SI. Hence, larger clusters rapidly complicate the analysis and would require vast computational efforts. Nevertheless, for smaller clusters, details about estimating *V*_C_ and *r̅* for nanospheres are provided in the SI and this analysis suggests that there is likely to be a high degree of disorder in the arrangement of the NPs. Moreover, *r̅* values may be distinguishable from the scattering patterns that are converted to length scales within the sample.

**Table 1:** Cluster Configuration and Estimates of Mean Separation for 43 and 145 nm Spheres

# NPS	configuration	*r̅* (μm) for	
		*d̅* = 43 nm spheres	*d̅* = 145 nm spheres
1	single sphere	0.20 ± 0.02	0.68 ± 0.19
2	linear rod	0.30 ± 0.03	0.98 ± 0.28
3	equilateral triangular prism	0.47 ± 0.10	1.26 ± 0.33
	linear rod	0.27 ± 0.12	1.13 ± 0.32
4	triangular pyramid	0.59 ± 0.11	1.58 ± 0.44
	square box	0.50 ± 0.10	1.34 ± 0.40
	linear rod	0.37 ± 0.13	1.20 ± 0.39
5	triangular bipyramid	0.75 ± 0.13	2.00 ± 0.58
	square pyramid	0.51 ± 0.13	1.69 ± 0.52
	arrow shape	0.46 ± 0.16	1.54 ± 0.48
	linear rod	0.40 ± 0.15	1.34 ± 0.46
6	square bipyramid	0.80 ± 0.17	2.10 ± 0.60
	rectangular box	0.46 ± 0.15	1.54 ± 0.47
	double arrow shape	0.53 ± 0.12	1.78 ± 0.51
	doughnut structure	0.50 ± 0.13	1.65 ± 0.50
	pentagonal pyramid	0.64 ± 0.12	1.74 ± 0.50
	linear rod	0.47 ± 0.12	1.52 ± 0.47

The scattering angle, θ, is associated with the scattering-induced momentum change experienced by the scattered photon, , where  and  are the scattered and reflected wavevectors. Assuming azimuthal symmetry, *q*^⊥^ = (2π/λ) tan θ, where λ = 400 nm and *q*^⊥^ =  × *n̂* is the component along the plane of reflection.^41^ High-angle scattering is expected from smaller length scales (Λ) within the sample, where Λ can represent individual and cluster scattering, characterized by *d̅*, or multiple interference from separated scatterers, characterized by *r̅*. The distribution of length scales, *f*(Λ), emerges by taking a Fourier transform of the scattering pattern, *F*(*q*^⊥^), such that
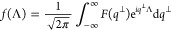
1

**Figure 4:**
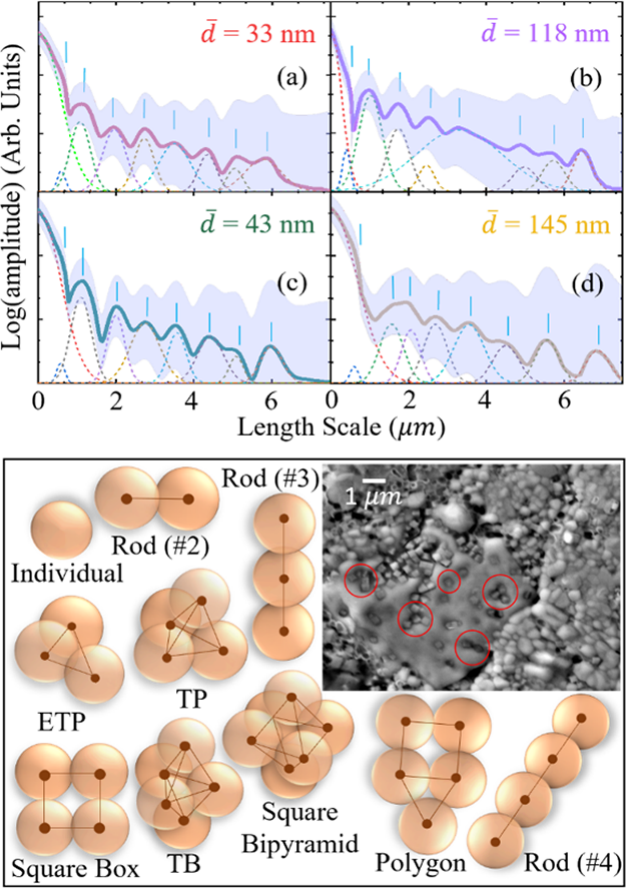
Mean separation and configuration of highly scattering small clusters: Scattering distribution *f*(Λ) as a function of the characteristic length scale within the disk, Λ, is acquired by a Fourier transform of scattering distribution as a function of angle. Scattering distributions are shown for (a) 33 nm and (b) 118 nm cubes and (c) 43 nm and (d) 145 nm spheres. The gray bands are confidence intervals, the peaks below the distribution are a series of Gaussian fits, whose peaks are marked by the vertical lines. (e) Example of cluster configurations used to estimate the mean separation between clusters (TP = triangular pyramidal, ETP = equilateral triangular prism, TB = triangular bipyramidal). The inset shows an electron micrograph of the KBr disk with 331 nm nanocubes illustrating the presence of a cluster of all different particle counts. The red circles highlight individual particles, two-particle rods, equilateral triangular prisms, and other 4-particle configurations not listed above.

Figure 4 shows *f*(Λ), the resulting numerical Fourier transforms of the scatter patterns from Figure 3, for (a) 33 nm and (b) 118 nm nanocubes and (c) 43 nm and (d) 145 nm nanospheres samples. The Fourier transforms of the scattering patterns are plotted on semilog plots to de-emphasize the strong peak at zero microns with respect to the weaker peaks at higher Λ. Each *f*(Λ) is fitted with a series of Gaussians to determine peaks for comparison to the estimated mean separation values.

Comparing Figure 4c with the *r̅* values in Table 1 for *d̅* = 43 nm nanospheres, it is seen that all values up to six-NP clusters remain at, or below, the first shoulder (centered at Λ ≈ 0.58 μm) of the zero-micron peak. Similarly, in Figure 4a, the first shoulder of the zero-micrometer peak of the *d̅* = 33 nm nanocubes sample occurs at Λ ≈ 0.59 μm. In these two cases, integrating *f*(Λ) gives ≈80% of the scatter comes from clusters with six or fewer NPs. Nevertheless, there is more structure in *f*(Λ) up to Λ ≈ 6 μm for both the small-NP samples. This indicates that there must be clusters that are significantly larger than six NPs. Alternatively, multiple scattering events or scattering from the next-nearest neighbor cannot be completely ruled out. Nonetheless, for individual small particles, *r̅* ≈ 4.7*d̅* because the NP concentration is ∝ Ø (= constant).

On the other hand, comparing Figure 4d with the *r̅* values in Table 1 for *d̅* = 145 nm nanospheres samples, the shoulder of the zero-micron peak occurs at Λ ≈ 0.6 μm. This corresponds well with the estimated *r̅* values for individually dispersed NPs in the sample. Similarly, the peaks at Λ ≈ 1.56 and 2.06 μm may correspond to the estimates of *r̅* arising from four-, five-, and six-sized-NP clusters. Overall, the large nanocubes samples give similar results. In these two cases, integrating *f*(Λ) gives ≈68% of the scatter comes from individual nanoparticles or small clusters, but there is more weight at higher Λ. As with small-NP samples, *f*(Λ) exhibits structure to higher values (up to Λ ≈ 7 μm), indicating even larger clusters.

Even from this basic analysis, it can be concluded that clustering is occurring despite errors introduced in the simplification of the shapes and the decoupling of *r̅* and *d̅* for samples with a large distribution of cluster sizes. Nevertheless, clustering is confirmed by an electron micrograph in Figure 4e that shows the surface of a KBr disk with large nanocubes dispersed in it and where individual and clustered NPs are visible. It should also be noted that clustering is not believed to be the result of creating the KBr-NP disks and is instead expected to be a result of the preparation of the NPs themselves since the electron micrographs of the NPs also exhibit clustering; see the insets of Figure 1. The wide range of cluster sizes makes quantitative analysis extremely challenging. Nevertheless, clustering may affect photocarrier dynamics.

### 3.2 Photocarrier Dynamics

Scattering of the pump photons means that the use of bulk absorption coefficients and Fresnel loss is insufficient to convert the incidence irradiance into the initial photoexcited carrier density in the transient absorption data. Hence, scattering affects the analysis of photocarrier dynamics and the extraction of mechanistic information provided by the inversion analysis. Calibration of the initial photoexcited carrier density in transient absorption data is easily overcome by measuring the irradiance of incident, transmitted, reflected, and scattered photons.

**Figure 5:**
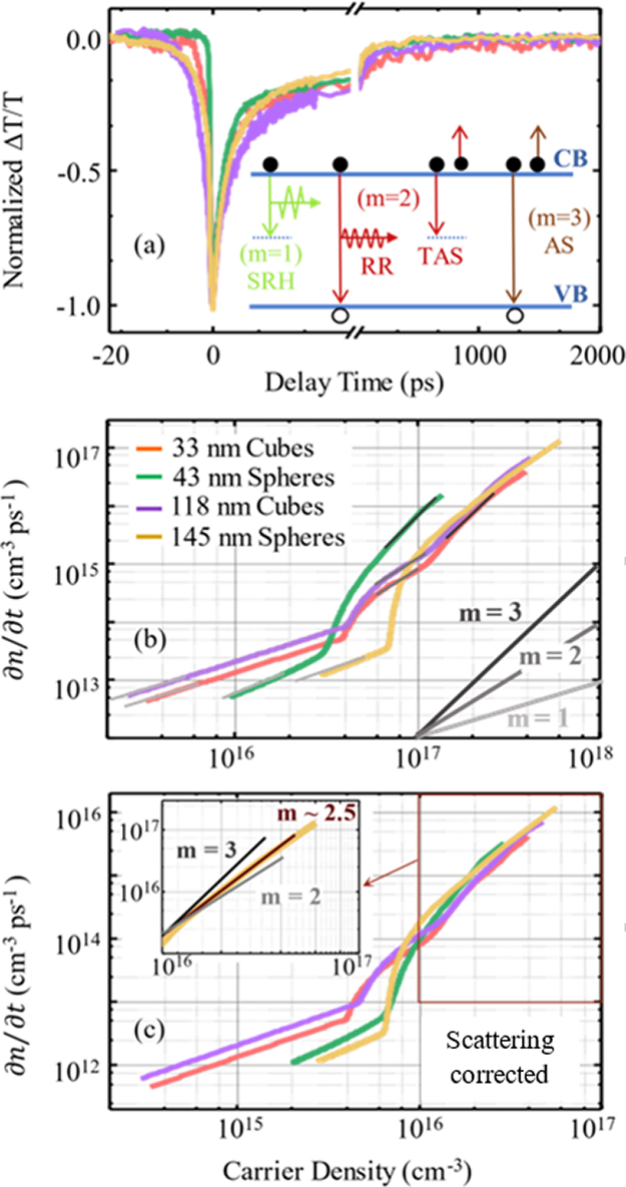
Transient absorption and photocarrier rate dynamics: (a) normalized transient absorption (Δ*T*/*T*) of 33, 43, 118, and 145 nm Cu_2_O nanoparticles. The inset shows schematic diagrams of the common recombination mechanisms, where Shockley-Read-Hall (SRH), radiative recombination (RR), trap-assisted Auger scattering (TAS), and Auger scattering (AS), respectively. Rate equations are determined from the transient absorption. Photocarrier population decay rate (∂*n*/∂*t*) as a function of photocarrier density (*n*) are shown (b) before and (c) after correcting for the scattered pump photons. In (b), slope guides are shown related to mechanisms shown in (a). Namely, slope *m* = 1 corresponds to SRH dynamics, *m* = 2 corresponds to RR or TAS, and *m* = 3 corresponds to AS. The rate curves in (b, c) are annotated with matching slopes to identify the mechanism. The inset of (c) shows an enlargement of the high-injection region of the rate curve for the 145 nm nanosphere sample to highlight the slope.

Figure 5a shows normalized differential transient transmission [Δ*T*/*T*]/|Δ*T*/*T*|_*t*=0_ from the KBr disks consisting of 33 and 118 nm nanocubes and 43 and 145 nm nanospheres. The data show the strongest signal near zero delay time (*t* = 0), with a fast decay at negative delay times (*t* < 0) and a protracted decay at positive delay times (*t* > 0) as the photocarrier population thermalizes and recombines. Specifically, the samples comprising larger NPs exhibit perturbed free induction for *t* < 0, arising from internal Mie scattering that leads to a coherent energy transfer between modes, as discussed previously.^18^ Such coherent phenomenon is absent in samples with small NPs.^34^ Near *t* = 0, the greatest differential transmission [Δ*T*/*T*]_*t*=0_ is approximately the same for the larger-NP samples and is 1.6× larger than that for smaller-NP samples. The relative strength of [Δ*T*/T]_*t*=0_ is likely due to the degree of scattering of the different particle sizes and is the first indication that scattering must be accounted for in calibrating and interpreting the transient data.

Using global analysis, a three-component multiexponential fit parametrizes the transient data and gives average lifetimes for each of the three components, τ_*m*_, where *m* = 1, 2, 3. For all four samples, these average lifetimes are ≈1, ≈30, and ≈1000 ps for the fast, intermediate, and slow decay components. Each component is dominated by a photocarrier recombination mechanism, such as those illustrated in the inset of Figure 5a. Each mechanism depends on the instantaneous photocarrier density, *n*, and the allowed interactions arising from the intra- and interband interactions, as the photocarriers thermalize and recombine. Hence, the transient data is a solution to a population rate equation,
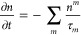
2where *n* and τ_*m*_ vary continuously with delay time. The rate equation can be recaptured from the transient amplitude by converting the data to *n*(*t*) and differentiating. In this case, the time-dependent photocarrier density is *n*(*t*) = [ – ln (| Δ*T*(*t*)/*T* |)]/(σ*l*), where *l* is the sample thickness and σ is the absorption cross-section, which reflects the conversion of photon density inside the sample to photocarrier density. The absorption cross-section is determined from the slope when plotting *n* ≈ *I*_0_(1 – *R*)α/*ℏ*ω_p_ versus σ*n* = *l*^–1^|Δ*T*(*t*)/*T*|_max_ for different incident pump irradiance, *I*_0_, and where *R* and α are the reflectivity and absorption coefficient of the sample; see Figure S4 of the SI for determination of σ. (Note that even in nonscattering samples, *R* and α may need to be evaluated with an effective medium theory.) Once calibrated, the recaptured rate equation describes only the recombination dynamics and is independent of the excitation mechanism. Nevertheless, the pump-irradiance dependence confirms that here only the above-gap single-photon was responsible for producing the photocarriers.

Figure 5 shows ∂*n*/∂*t* as a function of *n* for the four NP samples. On the right-hand side of the figure are color-coded slope guides for common photocarrier recombination mechanisms. These are illustrated in the inset of Figure 5a. A slope of *m* = 1 corresponds to SRH dynamics,^42^ where a carrier nonradiatively interacts with defect or other states whose population (or vacancy) is not a result of the optical excitation. A slope of *m* = 2 corresponds to RR or trap-assisted Auger scattering (TAS), where the former involves the bimolecular recombination of photoelectrons and photoholes. Similarly, the latter involves two photocarriers of the same species, where one recombines with a trap state and the resulting energy promotes the other deeper into its band through a free-carrier absorption process.^10^ Finally, a slope of *m* = 3 corresponds to AS, where a photoexcited electron–hole pair recombines, promoting a third photocarrier deeper into its band.^15^ The slope guides are used to highlight the dominant mechanism in various regions of the rate curves.

At low photocarrier densities (from *n* < 3 × 10^16^ cm^–3^ for 43 nm sphere to *n* < 7 × 10^16^ cm^–3^ for 145 nm sphere), all four samples exhibit SRH dynamics. Above this region, the dynamics are sample-dependent. Small spheres slowly transition to a high-photocarrier regime characterized by AS with a slope of *m* = 3, whereas large spheres transition to a slope of *m* ≈ 2.5, which is an admixture of AS and *m* = 2 processes such as RR and TAS; see the inset of Figure 5c. This slope indicates that the nanoparticles likely have traps and that in high photocarrier density regimes, Auger-like processes are prevalent. By contrast, nanocube samples exhibit transitions to an *m* = 2 regime, followed by further transition to a *m* = 3 region, suggesting that there are even more nonradiative mechanisms associated with the shape of the NPs. The second indication of the need to consider scattering is the wide range of *n* values for the transitions from SRH dynamics to higher slope mechanisms.

In scattering samples, the reflected scatter reduces the effective number of photons inside the sample to be absorbed. Hence, *n* ≈ *I*_0_(1 – η)α/*ℏ*ω_p_, where η = *R* + *S* is the extinction and *S* is the scattering amplitude, both of which are unitless. For the KBr disks consisting of 33 and 118 nm nanocubes and 43 and 145 nm nanospheres, it measured that η ≈ 25, ≈35, ≈26, and ≈32%, respectively. This indicated that larger NPs scatter more. In each case, plotting *n* versus σ*n* for various values of *I*_0_ will result in modified values of the σ value (see SI), which will alter the calibration of *n*(*t*) prior to differentiation. Without the correct evaluation of σ, the conversion of the transient data will lead to an overestimate of *n* and a warping of ∂*n*(*n*)/∂*t*.

Figure 5c shows the rate of decay of photoexcited carriers considering the scatter-corrected σ values. The observed changes include an overall reduction in the carrier concentration by an order of magnitude. Additionally, the shift is not uniform for each sample, resulting in a grouping of the rate curves by particle shape and not particle size. Now the nanocube samples exhibit a lower transition from the SRH regime to higher-order photocarrier dynamics as compared to nanospheres, which may be due to higher symmetry in spheres. Moreover, the smaller nanocube sample also exhibits a lower transition to higher-order photocarrier dynamics compared to the larger nanocube sample, which is consistent with increasing charge density in a smaller NP. On the other hand, the smaller nanocubes exhibit a lower SRH rate than the larger nanocubes, which is inconsistent with photocarrier density alone and may indicate that there are fewer nonradiative pathways for photocarrier recombination than in the larger particles. The nanocube samples exhibit *m* = 2 regions, which may be a result of TAS processes. However, this is circumstantial, and alternative measurements would be required to understand this behavior conclusively. By contrast, while the nanosphere samples have much lower SRH rates than the nanocubes overall, the smaller nanosphere sample exhibits rates lower than those of the small nanosphere sample, which would be consistent with a photocarrier-density effect. Moreover, the transition to higher photocarrier dynamics is approximately the same for both of the nanosphere-based samples. Finally, the rate for the various higher-order photocarrier dynamics, regardless of the mechanism, is approximately equivalent for all four samples. This result indicates that the photocarrier density is high, with respect to the particle size, even in the larger particles. Although the photoexcited carrier density reduces by nearly 1 order of magnitude, the mechanisms determined in both the low and high photocarrier density regimes remain unchanged, and thus initial determination of the dynamics mechanisms is also unchanged.

Correcting for scatter helps better estimate the photocarrier density and the resulting dynamics. For example, the spontaneous carrier recombination lifetime τ = *n*/(∂*n*/∂*t*) can now accurately be determined and compared between samples of different NP shapes and sizes; see Figure S5 of the SI. Additionally, the scatter pattern is informative of the clustering of NPs embedded in KBr disks. In the current sample set, the former presents dependence on the shape and exhibits similarities in the dynamics for samples consisting of NPs with the same shape. In contrast, the results of the scattering indicate a closer similarity with the size rather than shape. Nevertheless, both the photophysics and the distribution of NPs in an application will influence catalysts’ performance. In principle, there are competing design requirements that can be affected by the individual NP size and by clustering. In a low injection limit, excited by weaker light conditions, prolonged photocarrier lifetimes may be expected in nanocube samples. A high surface-to-volume ratio is desired for photocatalysis such that excessive clustering would effectively decrease it and be expected to reduce photocatalytic activity. On the other hand, clustering may also act like molecule traps between clustered nanoparticles to increase the dwell time of reagents inside the clusters and hence the effective activity.^27^ Moreover, if clusters are tightly packed this can enhance photocarrier exchange between NPs to improve charge separation, which also results in increased activity.^28,29^ This should inspire measurements of photocarrier dynamics with scatter correction for a series of samples where the NPs clustering is controlled. Clustering would also be expected to modify the Mie resonances, leading to a range of electrodynamics phenomena that could be tailored for application.^30,43,44^

## 4 Conclusions

In summary, Cu_2_O nanoparticles that have been identified for good photocatalytic activity due to Mie scattering resonances have been investigated for their charge carrier dynamics. To accurately estimate the photocarrier density in such measurements, the sample nanoparticles were compressed into a KBr disk, during which time Cu_2_O nanoparticle clusters were embedded into the host KBr matrix. The resulting samples are highly scattering, such that the optical measurements must consider not only the reflection and transmission of the optical excitation but also the pump scattering by taking into account the full extinction of the pump light at the incident surface of the sample. In doing so, the estimated photocarrier density is reduced by approximately 1 order of magnitude for samples comprised of nanoparticles with sphere and cube shapes and ranging of characteristic length scale (30 nm < *d̅* < 145 nm). However, the extracted mechanisms in the charge carrier dynamics do not appear to be changed by the correction. Nevertheless, the pump-scatter correction allows for better comparison in the range of photocarrier density where certain mechanism dominates the dynamics. In this case, comparing the measured range where Shockley-Read-Hall dynamics dominates the dynamics, it is seen that particle shape, rather than its size, limits the upper bound of this low-photocarrier-injection regime. Above this regime, the instantaneous rate is almost identical–independent of the photocarrier recombination mechanism, or particle shape or size. Both these findings are somewhat unexpected because the size and shape of nanoparticles have typically been thought to strongly dominate the photocarrier dynamics and extracted rates.^45,46^

During the process of correcting the photocarrier density, the pump-scatter pattern can be measured and mapped through a Fourier transform to a distribution of characteristic length scales within the sample. The distribution of characteristic length scales represents the distribution of mean sizes of and separations between scatterers. As is shown, the latter can be estimated based on the known volume ratio of Cu_2_O nanoparticles and KBr host, and by simplifying the cluster shapes and volumes. This approach reveals that for distributions of larger nanoparticles small clusters are resolvable–even with a short distance between the samples and the view screen. Moreover, the pump scattering is strong for all nanoparticles and all the measured samples exhibit clustering. This is confirmed by electron micrographs for all Cu_2_O nanoparticles in powders and the KBr disks. More detailed analysis of smaller clusters and individual scatterers can be performed by looking at small scattering angles and estimates of larger cluster volumes can be achieved through more sophisticated computing approaches. For current samples, the inhomogeneity of the sample makes a firm prediction of the cluster sizes somewhat crude, which may perhaps be overcome by fabricating nanoparticles with surfactant coatings that alter their stickiness and minimize the range of cluster sizes that are formed, both in solution and in disk form used in optical experiments. A systematic study would be needed to properly control the cluster size and its relation to both the mean separation of clusters in these optical samples and any changes in the photocarrier rate dynamics. Nonetheless, this study offers links to scattering and photocarrier dynamics in real photocatalysts and provides a recipe for the accurate quantification of the photocarrier dynamics.

## Supporting Information Available

Detailed descriptions of photocatalyst synthesis followed by their corresponding FDTD simulations; clusters’ volume calculation procedure and table for calculated volume; plot showing dispersion in mean separation values with respect to the number of NPs in a cluster; plots for determining absorption cross-section for rate equation analysis and the spontaneous carrier recombination lifetime; and parameters from multi-Gaussian fit (PDF)
