# Supplementary material for: Photocarrier Recombination Dynamics in Highly Scattering Cu2O Nanocatalyst Clusters
Source: J Phys Chem C Nanomater Interfaces. 2024 Jan 24;128(5):2003–11. doi: 10.1021/acs.jpcc.3c06941 (PMC10860136; doi:10.1021/acs.jpcc.3c06941)
Supplement: Supplementary file 1 — jp3c06941_si_001.pdf [file jp3c06941_si_001.pdf]

Photocarrier Recombination Dynamics in Highly Scattering Cu_2O Nanocatalysts Clusters

Sunil Gyawali,[1] Ravi Teja A. Tirumala,[2] Harrison Loh,[3] Marimuthu Andiappan,[2] Alan D. Bristow[1]

[1] *Department of Physics and Astronomy, West Virginia University, Morgantown, WV 26506, USA.*
[2] *School of Chemical Engineering, Oklahoma State University, Stillwater, OK 74078, USA.*
[3] *Department of Mechanical and Aerospace Engineering, West Virginia University, Morgantown, WV 26506, USA.*

Supporting Information (SI):

1. Syntheses of Cu_2O Nanospheres and Nanocubes

1.1. Cu_2O Nanospheres:

Smaller Cu_2O nanospheres, with diameters ranging from 35 to 45 nm, were synthesized utilizing the microemulsion technique at room temperature (around 20°C). In this specific synthesis process, a 250 mL round bottom flask was employed. Within this flask, a mixture of 54.5 mL of n-heptane (serving as the oil phase) and polyethylene glycol-dodecyl ether (Brij, having an average Mn of approximately 362) was introduced. The entire mixture was then stirred at a speed of 550 revolutions per minute (rpm). A solution consisting of 5.4 mL of 0.1 M copper(II) nitrate in an aqueous medium was introduced into the aforementioned mixture. Additionally, a 1 M solution of hydrazine (5.4 mL) was added as the reducing agent. The microemulsion was allowed to stir for a span of 12 hours. Subsequent to this duration, acetone was added to the mixture to disrupt the emulsion. The resulting mixture was then subjected to centrifugation. The nanoparticles produced in this process were subjected to a thorough washing regimen, involving three iterations of washing. Each washing cycle encompassed sonication followed by centrifugation, aimed at eliminating the surfactant and yielding Cu_2O spherical nanoparticles.

The synthesis of larger Cu_2O nanospheres, with a diameter of approximately ~145 nm, was achieved through the utilization of the chemical reduction method at a synthesis temperature of 55°C. Initially, a 100 mL round bottom flask was employed to prepare a 10 mM $CuCl_2$ aqueous solution, consisting of 50 mL. The mixture was subjected to stirring at a rate of 900 revolutions per minute (rpm) while maintaining a temperature of 55°C. Subsequently, a 2 M NaOH solution of 5 mL was introduced into the mixture. This combined mixture continued to stir under consistent heating at 55°C for a duration of 30 minutes. Following this, an additional 5 mL of a 0.6 M ascorbic acid aqueous solution, serving as the reducing agent, was added to the mixture. The synthesis mixture underwent stirring for a period of 5 hours. The resultant nanoparticles were separated from the synthesis mixture through a thorough washing process. This entailed washing the nanoparticles three times in both deionized (DI) water and ethanol. The aim of this washing was to meticulously remove any residues associated with the synthesis mixture.

1.2. Cu_2O Nanocubes:

Using a chemical reduction method at room temperature (around 20°C), we successfully synthesized larger Cu_2O nanocubes with average edge lengths ranging from 280 to 300 nm. The synthesis process involved several key steps. Prepare a copper source through the combination of 30 mL of a 0.0032 M aqueous $CuCl_2$ solution. This solution was carefully introduced into a three-neck round bottom flask placed within an inert nitrogen environment. At room temperature, we added 1 mL of a 0.35 M aqueous NaOH solution to the

$CuCl_2$ mixture, promptly leading to the creation of distinct, blue-colored $Cu(OH)_2$ colloids. Through gradual and incremental additions of sodium ascorbate (the reducing agent), the solution underwent a visible transformation to an orangish-yellow shade, signifying the formation of cubic Cu_2O particles. This synthesis phase extended for a period of one hour. Following synthesis, the Cu_2O nanocubes underwent a thorough washing process involving ethanol, with three cycles of washing that incorporated both sonication and subsequent centrifugation.

Small Cu_2O nanocubes with an edge length of approximately ~33 nm was synthesized through a chemical reduction method conducted at room temperature (around 20°C). In this process, a 120 mL aqueous solution of $CuCl_2$ with a concentration of 0.0032 M was introduced into a 250 mL three-neck round bottom flask. The flask was then placed within an environment saturated with inert nitrogen gas for a duration of 45 minutes. Following this, 4 mL of a 0.35 M aqueous NaOH solution was added, and subsequently, a solution containing 0.1 M of sodium ascorbate (acting as the reducing agent) was introduced with a volume of 4 mL. The resulting solution underwent a noticeable transformation to a vibrant yellow hue, indicating the formation of Cu_2O nanoparticles. These nanoparticles took the form of nanocubes with an edge length of 33 ± 6 nm. After the course of 45 minutes, the Cu_2O nanocubes were subjected to the same washing procedure as previously mentioned. The Cu_2O nanocubes underwent a thorough washing process involving ethanol, which was repeated three times. During each washing step, the nanocubes were subjected to sonication and subsequent centrifugation to ensure effective cleansing.

2. Finite difference time-domain simulations of the extinction spectra for nanoparticles

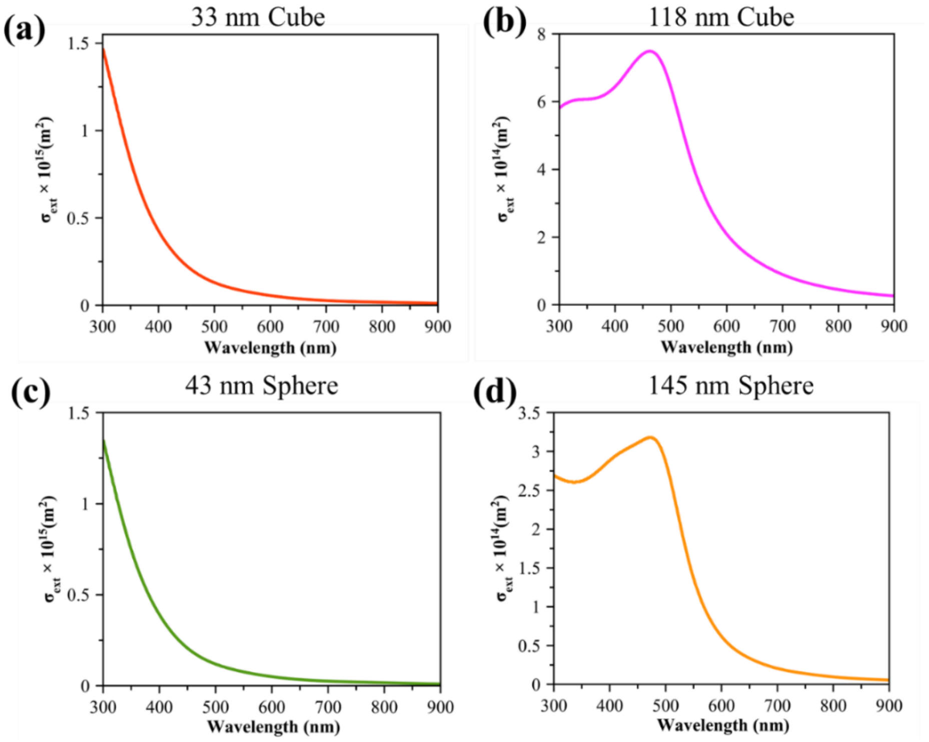

Fig. S1 Finite-difference time-domain simulations of the extinction spectra for Cu_2O nanocubes with average edge length (a) 33 nm, (b) 118 nm and nanosphere with average diameter (c) 43 nm, (d) 145 nm.

Figure S1 shows the finite-difference time-domain (FDTD) simulation for the four nanoparticle samples investigated in this work. The detailed procedure used for FDTD simulation are provided in our previous contribution [1].

3. Volume Calculations for Clusters

Figure S2 (a) shows the cartoon of the nanoparticle clusters (NPCs) embedded in KBr matrix to form a semi-transparent disk of diameter $\sim 1\ cm$ and the thickness of $\sim 0.5\ mm$. As shown in the figure, NPCs inside the sample disk may have different configuration that can be estimated from a Fourier transform of the scattering profile and matched approximately to the values obtained from the calculation of mean separation given by Table 1 in main manuscript.

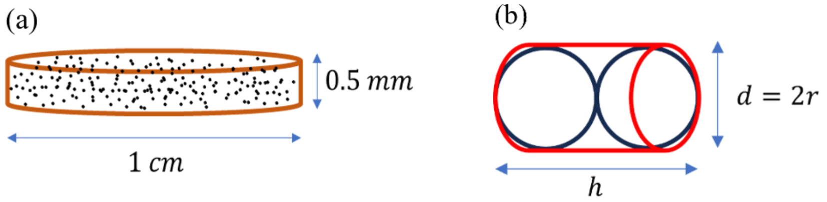

Fig. S2 (a) Nanoparticles dispersed inside the KBr matrix forming semi-transparent disks, (b)Volume estimation for clustering of two small spheres (43 nm) forming a linear rod.

Figure 2 (b) shows two spherical NP forming NPC that resemble a linear cylindrical rod assuming they are in contact with each other. We know that the volume of a cylindrical rod, $= \pi r^2 h = 1.25 \times 10^{-4}$ μm^3 for 43-nm spheres, where $r \approx 21.5$ nm and $h \approx 2 \times d = 86$ nm. In this similar manner, for different possible configurations the volume of the clusters is calculated for 43-nm and 145-nm spheres. Table S1 below gives the calculated values of volume of NPCs in different arrangements.

Table S1 shows a list of estimated V_C for small clusters comprised of 43-nm and 145-nm nanospheres.

# NPs	Configuration	V_c (μm^3) for	
		$\bar{d}$=43 nm spheres	$\bar{d}$=145 nm spheres
1	Single Sphere	$(4.00 \pm 1.20) \times 10^{-5}$	$(1.57 \pm 0.43) \times 10^{-3}$
2	Linear Rod	$(1.25 \pm 0.37) \times 10^{-4}$	$(4.70 \pm 1.34) \times 10^{-3}$
3	Equilateral Tri. Prism	$(5.19 \pm 3.30) \times 10^{-4}$	$(9.60 \pm 4.31) \times 10^{-3}$
	Linear Rod	$(9.84 \pm 4.12) \times 10^{-5}$	$(7.21 \pm 2.76) \times 10^{-3}$
4	Triangular Pyramid	$(1.02 \pm 0.53) \times 10^{-3}$	$(1.97 \pm 0.62) \times 10^{-2}$
	Square Box	$(6.25 \pm 3.75) \times 10^{-4}$	$(1.20 \pm 0.54) \times 10^{-2}$
	Linear Rod	$(2.53 \pm 1.06) \times 10^{-4}$	$(9.53 \pm 3.78) \times 10^{-3}$
5	Triangular Bipyramid	$(2.10 \pm 1.09) \times 10^{-3}$	$(4.00 \pm 1.86) \times 10^{-2}$
	Square Pyramid	$(6.63 \pm 3.07) \times 10^{-4}$	$(2.41 \pm 1.04) \times 10^{-2}$
	Arrow Shape	$(4.86 \pm 2.07) \times 10^{-4}$	$(1.82 \pm 0.79) \times 10^{-2}$
	Linear Rod	$(3.20 \pm 1.40) \times 10^{-4}$	$(1.20 \pm 0.58) \times 10^{-2}$
6	Square Bipyramid	$(2.56 \pm 1.63) \times 10^{-3}$	$(4.63 \pm 2.14) \times 10^{-2}$
	Rectangular Box	$(4.86 \pm 2.75) \times 10^{-4}$	$(1.82 \pm 0.93) \times 10^{-2}$
	Double Arrow Shape	$(7.44 \pm 4.05) \times 10^{-4}$	$(2.81 \pm 1.34) \times 10^{-2}$
	Doughnut Structure	$(6.25 \pm 4.07) \times 10^{-4}$	$(2.24 \pm 1.19) \times 10^{-2}$
	Pentagonal Pyramid	$(1.31 \pm 0.73) \times 10^{-3}$	$(2.63 \pm 1.42) \times 10^{-2}$
	Linear Rod	$(5.19 \pm 2.97) \times 10^{-4}$	$(1.75 \pm 0.87) \times 10^{-2}$

4. Dispersion of Mean Separation

Figure S3 shows the range of mean separation values that can be observed for different NPC with same number of NPs in the cluster for 145 nm spheres. Statistically, in a sample disk, the number of NPs will remain the same and will have some mean separation between them if they remain in single form. Now when NPs start forming clusters, there mean separation will start to increase.

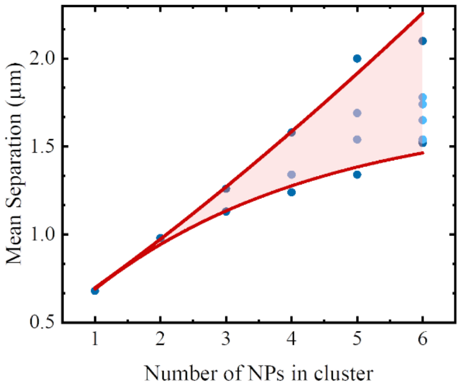

Fig. S3 Dispersion in the mean separation values of the clusters for increasing nanoparticles counts in nanoparticle clusters.

From the figure it is clear that as we increase the NPs numbers in clusters, their mean separation values start dispersing more and more. For example, if two NP forms cluster they could only be arranged such that they touch each other and thus we will have a single value of mean separation. And if we have 3 NPs, they can be arranged such that they can either form linear rod or a triangular prism such that we only have two values of mean separation. In the same way this value of mean separation starts dispersing more and more on increasing number of NP in cluster. This is true and can easily be imagined that if we go to more than 5 NPs in the clusters, the shape they form during cluster formation can be largely different in number. This is the reason we stopped calculating the volume of clusters after 6 number of NPs in clusters.

5. Determining the Absorption Cross-Section for Rate-Equation Analysis

To get the rate $\partial n/\partial t$, we first need to find the photocarrier density $n(t)$. It can be estimated using the relation $n(t) = [-\ln(|\Delta T(t)/T|)]/(\sigma l)$ where l is the sample thickness and σ is the absorption cross-section. Here, $\Delta T(t)/T$ is our experimental data and $l \sim 0.5$ mm. Differentiating $|\Delta T(t)/T|$ for $\partial n/\partial t$ is prone to noise. So, first we need to filter the transient data with multi exponential fitting (3 exponential in our case) before differentiating it. Now the only thing that needs to be calculated is σ. σ can be calculated from the slope when plotting $n \approx I_0(1-\eta)\alpha/\hbar\omega_p$ versus $\sigma n = l^{-1}|\Delta T(t)/T|_{max}$, where I_0 is the pump irradiance, which is related to the incident average power. Figure S4 shows n versus σn determined from the average power-dependent measurements to obtain the slope $|\Delta T(t)/T|_{max}$, and hence σ.

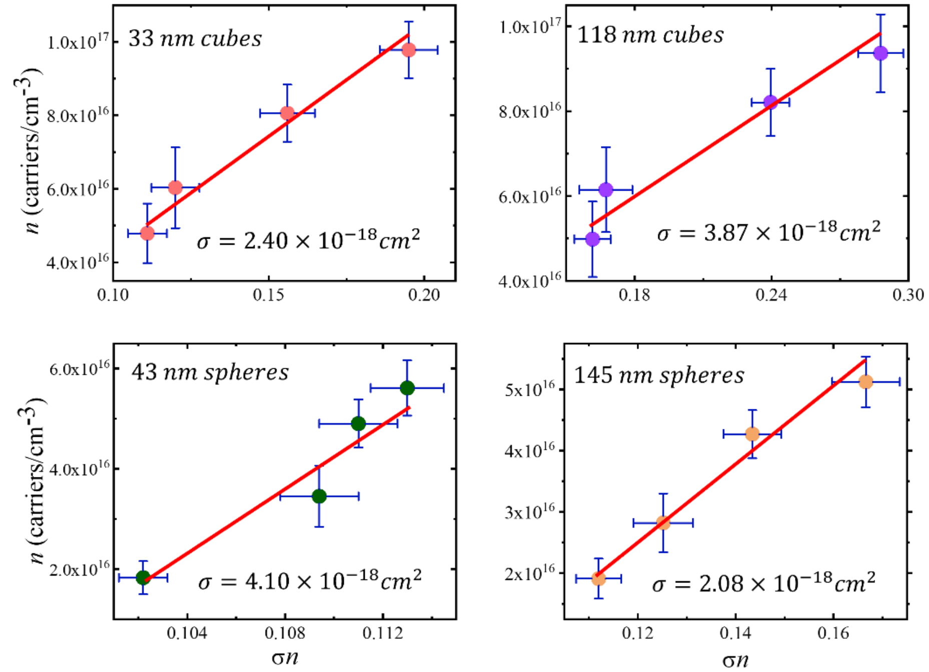

Fig. S4 Plot of n vs σn with calculated values of absorption cross-section for all four nanoparticles.

6. Spontaneous Carrier Recombination Lifetime

Now that the photocarrier density n and rate curves $\partial n/\partial t$ have been corrected for loss of pump photons due to scattering, the spontaneous carrier recombination lifetime $\tau(n) = n/(\partial n/\partial t)$ can be accurately determined and compared. Figure S5 shows $\tau(n)$ for all four samples.

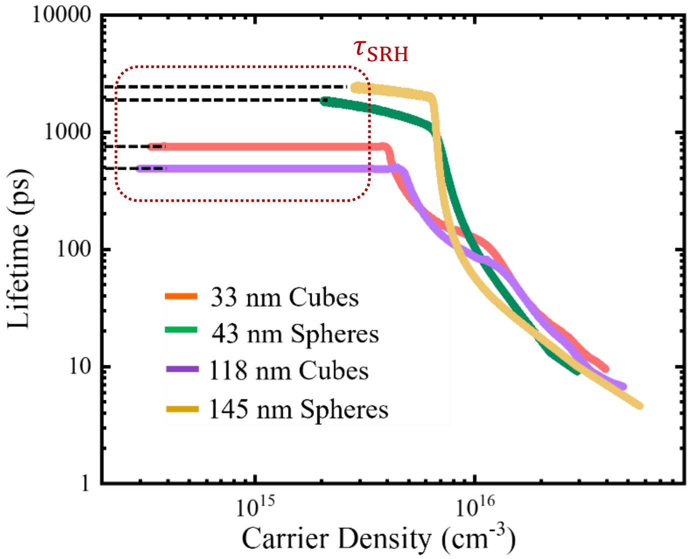

Fig. S5 Instantaneous photocarrier lifetime.

As is seen in the figure, each sample exhibits a distinct low-injection photocarrier density lifetime, which decreases with increasing photocarrier density as higher-order recombination mechanisms are activated. In the low injection range, the lifetimes are $\tau_{\mathrm{SRH}} \approx 800$ ps for small cubes, $\tau_{\mathrm{SRH}} \approx 600$ ps for larger cubes, $\tau_{\mathrm{SRH}} \approx 2000$ ps for small spheres, and $\tau_{\mathrm{SRH}} \approx 2200$ ps for large spheres. In contrast, the fast recombination lifetimes tend toward a similar value for a given photocarrier density for all four samples, even if the slope and mechanism may depend on size and shape.

7. Multi-Gaussian Fitting Parameters

Table S2 shows the fitting parameters for the 9 Gaussians used to best fit the scattering distribution, $f(\Lambda)$.

Table S2 Parameters for the Gaussians best fits to the scattering distribution, $f(\Lambda)$.

Amplitudes									
	1st	2nd	3rd	4th	5th	6th	7th	8th	9th
33 nm	2.69	0.365	1.20	1.077	0.91	0.825	0.64	0.40	0.55
43 nm	2.69	0.34	1.349	1.077	0.941	0.793	0.733	0.421	0.582
118 nm	2.715	0.72	1.64	1.07	0.448	1.054	0.416	0.530	0.691
145 nm	2.727	0.276	0.935	0.845	0.933	0.922	0.597	0.685	0.519
Mean Value (Λ_0)									
	1st	2nd	3rd	4th	5th	6th	7th	8th	9th
33 nm	0.0044	0.588	1.07	1.95	2.718	3.49	4.34	5.04	5.73
43 nm	0.029	0.58	1.10	2.00	2.747	3.56	4.42	5.08	6.00
118 nm	0.017	0.40	1.04	1.73	2.481	3.317	4.98	5.72	6.47
145 nm	0.008	0.0603	1.561	2.06	2.689	3.556	4.50	5.56	6.86
Gaussian Widths (Standard deviation)									
	1st	2nd	3rd	4th	5th	6th	7th	8th	9th
33 nm	0.534	0.10	0.25	0.30	0.252	0.40	0.23	0.19	0.46
43 nm	0.55	0.09	0.264	0.223	0.385	0.232	0.336	0.177	0.279
118 nm	0.262	0.09	0.30	0.28	0.199	1.096	0.311	0.289	0.229
145 nm	0.66	0.106	0.308	0.245	0.302	0.396	0.339	0.301	0.31

References

(1) Tirumala, R. T. A.; Gyawali, S.; Wheeler, A.; Ramakrishnan, S. B.; Sooriyagoda, R.; Mohammadparast, F.; Khatri, N.; Tan, S.; Kalkan, A. K.; Bristow, A. D.; Andiappan, M. Structure–Property–Performance Relationships of Cuprous Oxide Nanostructures for Dielectric Mie Resonance-Enhanced Photocatalysis. *ACS Catal.* **2022**, *12* (13), 7975–7985. https://doi.org/10.1021/acscatal.2c00977.
